# A multi-modal relative spatial access assessment approach to measure spatial accessibility to primary care providers

**DOI:** 10.1186/s12942-018-0153-9

**Published:** 2018-08-23

**Authors:** Yan Lin, Neng Wan, Sagert Sheets, Xi Gong, Angela Davies

**Affiliations:** 1 Department of Geography and Environmental Studies, 1 University of New Mexico, MSC 01 1110, Albuquerque, NM 87131 Mexico; 2 Department of Geography, University of Utah, 260 S. Central Campus Dr., Room 270, Salt Lake City, UT 84112-9155 USA

**Keywords:** 2SFCA, E2SFCA, Multi-modal, Gaussian function, Spatial access, Primary care

## Abstract

Two-step floating catchment area (2SFCA) methods that account for multiple transportation modes provide more realistic accessibility representation than single-mode methods. However, the use of the impedance coefficient in an impedance function (e.g., Gaussian function) introduces uncertainty to 2SFCA results. This paper proposes an enhancement to the multi-modal 2SFCA methods through incorporating the concept of a spatial access ratio (SPAR) for spatial access measurement. SPAR is the ratio of a given place’s access score to the mean of all access scores in the study area. An empirical study on spatial access to primary care physicians (PCPs) in the city of Albuquerque, NM, USA was conducted to evaluate the effectiveness of SPAR in addressing uncertainty introduced by the choice of the impedance coefficient in the classic Gaussian impedance function. We used ESRI StreetMap Premium and General Transit Specification Feed (GTFS) data to calculate the travel time to PCPs by car and bus. We first generated two spatial access scores—using different catchment sizes for car and bus, respectively—for each demanding population location: an accessibility score for car drivers and an accessibility score for bus riders. We then computed three corresponding spatial access ratios of the above scores for each population location. Sensitivity analysis results suggest that the spatial access scores vary significantly when using different impedance coefficients (p < 0.05); while SPAR remains stable (p = 1). Results from this paper suggest that a spatial access ratio can significantly reduce impedance coefficient-related uncertainties in multi-modal 2SFCA methods.

## Background

Primary health care has been a main focus of health care policy, provision, and research for 40 years—a focus that was reaffirmed by the World Health Organization in 2008 [47]. It is a movement that has been focused on comprehensive, continuous, and person-centered care; often described in contrast to care that is narrowly specialized, focused on short-term results, or fragmented in its delivery [47]. Primary health care commonly takes the form of generalist physicians who address the four main features of primary care: to be the first contact for new needs; to provide long-term person-focused care (as opposed to disease-focused); to offer comprehensive care for most health needs; and to coordinate specialist care when needed [36]. As a result, primary care is associated with greater access to services, higher quality of care, a focus on prevention, early management of health problems, the cumulative effect of these qualities, and a reduction in unnecessary specialist care [36, 51].

At the population level, strong primary care is associated with better health and slower growth in health care spending [17], as well as lower rates of avoidable hospitalizations [17, 31]). In some specific examples, primary care, with its dimensions of continuous, coordinated, and comprehensive care, is considered a key element of cancer control (including in prevention, diagnosis, survivorship, and end-of-life care) [23, 25, 32] and is associated with better self-rated health in people with chronic conditions [11].

### Access to primary health care

Access is considered a basic tenet of primary care, particularly as it relates to first contact (between providers and patients) [34]. In addition, it can be considered a benefit of primary care [35], a measure of primary care [31], and a mechanism by which population health benefits [36]. However, a specific definition of access and description of its role in primary health care has been elusive. It has been defined and studied as: availability and volume of services; geographic accessibility in terms of travel distance; accommodation of accessibility, such as appointment systems and operating hours; affordability; acceptability and patient satisfaction; utilization, or actual consumption of services; and equality in access [4, 18].

The above discussion is not limited to research on primary health care. In research on access to health care in general, access has been described in terms of both the characteristics of the health care delivery system and the characteristics of the population of interest. There has been a particular focus on access as an interaction or fit between the system and population (utilization of services) and the health outcomes that result from this interaction [1, 30].

Access to primary care physicians has been refined into a framework of potential and realized (sometimes “revealed”) access. Realized access includes the actual rates of consumption and the reported descriptions of care received, and can be measured objectively (utilization) or subjectively (customer satisfaction). Potential access describes the organization and capabilities of the health care system (such as facilities, doctors, and costs) and the potential of the consumers (including wants, needs, and resources) [2, 3].

As health systems have grown more complicated, so have dimensions of access, with recent models accounting for approachability, acceptability, availability and accommodation, affordability, and appropriateness of services, as well as patients’ abilities to perceive, seek, reach, pay, and engage [22]. However, some common themes emerge across conceptions and models. Geography, spatial configuration, and mobility are examples, as seen in a patient’s “ability to seek” [22]. Travel time has been identified as a strong predictor of satisfaction with accessibility and associated with opportunity cost for service use [24, 30].

The influence of geography has been made explicit in some taxonomies as researchers attempt to organize the definitions of access. Khan and Bhardwaj [16] took the stages of access—potential and realized—and added two crosswise dimensions: spatial (geographic) and aspatial (social, economic, and cultural). This taxonomy has become a central paradigm in geographic studies of healthcare access, as it makes clear the strengths and limitations of available data and the analyses performed [9, 15, 16].

### Potential spatial access: previous approaches

Studies of “potential spatial” access to health care resources have maintained a focus on the spatial interaction of providers and populations, particularly with gravity models, which model the potential spatial interaction between supply and demand and focus on how distance affects the attraction of a supply or service and the cost for those who demand it [9]. These models are more sophisticated measures of access than previous measures of regional availability, which were ratios of health care resources to populations within a predefined regional unit (e.g., census tract) [26].

#### Single mode approaches

Floating catchment area models (FCA) are a relatively recent development in measures of potential spatial access. In particular, the two-step floating catchment area model (2SFCA) (e.g., [27, 45]) has been widely used due to its ability to assess potential spatial access where realized access information is unavailable, its relative ease of implementation, and its conceptual completeness [26]. “Catchment area” refers to both the service area of a resource or supply point and the area of opportunities for a population center, both measured in travel time. Catchment size, unlike regional or administrative units, may be adjusted for the attraction of the resource or the abilities of the population; their boundaries “float.”

However, this model is limited in its ability to account for distance decay. All populations within the service area are assigned an equal access score, regardless of differences in proximity or travel time to a service. More recent permutations of 2SFCA models have addressed this weakness in different ways, and the enhanced two-step floating catchment area method (E2SFCA) was one of the first to explicitly employ a travel impedance function. Catchments are divided into travel-time subzones and opportunities within each subzone are weighted by a mathematical function of travel impedance. In other words, populations near a resource may receive a weight near 1, while populations near the edges of the catchment receive a weight closer to 0. The complete process of this model is discussed in the “Review of E2SFCA and the relative spatial access assessment method” section.

Two limitations of concern have emerged for the E2SFCA method: (1) high variations in access scores, introducing uncertainty as to whether scores are attributed to the choice of the impedance coefficient rather than the configuration of the system, and (2) it only allows for one mode of transportation, and studies have generally used personal automobiles to establish travel times. The first concern has been addressed, in part, by the introduction of a spatial access ratio (SPAR), that normalizes the access scores (spatial access indices, or SPAI) by the average score of the area of interest [42]. SPAR is discussed in greater detail in the “Review of E2SFCA and the relative spatial access assessment method” section. The current research seeks to address both concerns, and here we present a review of other studies that have examined multi-modal access.

#### Multi-modal approaches

Multi-modal approaches are methods that account for differences in modes of transportation. Privately owned automobiles (cars) and public transportation, for example, have been shown to have different accessibility indices. In other words, different modes of transportation allow their users differing levels of access to resources [20]. This finding—particularly that cars provide greater spatial access than public transportation—has held true across computational models [33]. Much of the research on accessibility variations in transport modes has been undertaken in the aim of studying sustainable development and equitable access, and the gap has been found to persist even as reforms are implemented to improve public transit [5].

Studies on multi-modal accessibility are scarce compared with those on single-modal accessibility and focus primarily on measuring and comparing accessibility by different transportation modes to places that represent health behaviors. For example, a study of park access found that in addition to public transit, bicycle and walking modes reduce accessibility compared to cars [50]. In another case, accessibility to supermarkets was higher for cars than public transit, and that accessibility scores for public transport had greater variability [48]. However, a study of access to healthy food found that public transport offers greater accessibility to some resources at certain times of day [38]. A statistical analysis of transportation modes and cancer screening in England found that car ownership was strongly associated with screenings for breast and cervical cancer, while public transit use was inversely associated with breast cancer screenings [44]. Finally, Higgs et al. [12] used single-mode E2SFCA scores from independent transportation networks (car and bus) as inputs in a multivariate analysis of general practitioner access and observed that bus scores were lower than car [12]. However, these studies were still based on single-modal methods to measure spatial access under each transportation mode separately.

Several studies developed new multi-modal methods using the FCA framework with multiple transportation modes. Mao and Nekorchuk [28] investigated accessibility to healthcare in Florida. With the 2SFCA model as a foundation, they added a multi-modal element to address the assumption that all populations within a catchment have equal access. Populations were divided into subpopulations based on census data on vehicle ownership at the block group level and multiple catchments were created at each healthcare location, one per transportation mode. Weights were applied based on the sizes of the subpopulations in their service areas. The analysis was run for the entire state, and they found that an unmodified 2SFCA resulted in higher accessibility scores than the multi-modal method. This model incorporated both travel modes into one score rather than comparing car and bus scores. Bus routes were not used; as a proxy, while buses travelled the same network as cars, they did so at a slower speed [28].

Dony et al. [7] researched access to parks in Mecklenburg County, North Carolina and introduced a variable-width floating catchment area (VFCA), in which catchment size varies with a measure of park attractiveness and with a travel mode coefficient. In the modified second step, park-to-population ratios were weighted by the distance from the population to the park. Spatial accessibility scores were calculated once per mode of transport using a Google Maps API, which makes some consideration of the best network or path for each mode (e.g., directing cyclists to designated bike paths). In their case study, car, bus, bicycle, and walking were compared, and the VFCA method was compared to 2SFCA scores derived with fixed catchment sizes. The VFCA scores showed greater variability and walking resulted in low scores across models [7].

Langford et al. [21] introduced modifications to the 2SFCA model to produce separate accessibility scores for each mode and demonstrated the model with a case study of access to primary health care in South Wales, UK. The researchers limited bus access to bus stops and used an independent bus route network, and the modified model allows for additional unique networks. A single access score was still generated for each supply location, but it was based on the combined population using the modeled travel modes. In the second step, a separate score was generated for each mode at each population location. Subpopulations were established by census data on car ownership. They compared car-only E2SFCA scores to multi-modal scores and found that bus riders experience much lower accessibility. This also meant that car access scores in the multi-modal model were higher than in the single-mode model.

Xing et al. [49] also investigated access to parks with a multi-modal 2SFCA model and a case study in Wuhan, China. Populations were assigned a travel mode based on their proximity to parks and travel modes were assigned different speeds on one network to determine travel time. This produced an integrated accessibility score that was compared to single-mode 2SFCA scores [49].

Finally, Tao et al. [37] employed two independent online map APIs for different travel modes to measure accessibility to healthcare services in Shenzhen, China. The two Baidu Map services were used to estimate car travel times and public transit travel times separately, and the APIs restrict public transit to designated routes. Their model allowed for the calculation of both integrated and mode-specific accessibility scores, and comparisons to the conventional, single-mode 2SFCA showed that the multi-modal method revealed more disparities in access. Specifically, transit-reliant populations were more disadvantaged [37].

These studies are distinguished by their use of networks or travel-speed proxies to model differences in travel modes. Two studies used the same networks for all modes, differentiating between them by adjusting the speed for each mode, which is recognized as a limitation since many modes of travel have restricted or unique routes, such as bus routes or bike paths [28, 49]. Two studies utilized online mapping services to address this limitation and route travelers on an appropriate network for their travel mode [7, 37], while one used two independent networks in a desktop GIS environment [21].

Although multi-modal two-step floating catchment area methods provide more realistic accessibility representation than single-modal methods, the use of the impedance coefficient in the weighting function (e.g. Gaussian function) and the associated variations in the accessibility scores remains a source of uncertainty. Uncertainty is a state of limited knowledge which causes difficulties in exactly describing an outcome or more than one outcome. In the two-step floating catchment area methods, uncertainties arise due to the lack of knowledge about impedance coefficients, which lead to difficulties in exactly quantifying the spatial access scores. This paper therefore proposes an enhancement to the multi-modal two-step floating catchment area methods through incorporating the spatial access ratio (SPAR) for spatial access measurement. An empirical study on spatial access to primary care physicians in the city of Albuquerque, NM, USA was conducted to evaluate the effectiveness of SPAR in addressing uncertainty introduced by the use of different impedance coefficients in the classic Gaussian impedance function.

## Methods

We propose our multi-modal relative spatial access assessment approach based on the E2SFCA method and the relative spatial access assessment method.

### Review of E2SFCA and the relative spatial access assessment method

There are two steps in the E2SFCA method. The travel-time measurements may vary by resource or study area, but the original method uses the common 30-min catchment. In the first step, the model defines a 30-min travel-time zone (catchment) for each healthcare location *i*, and divides the travel-time zone into three subzones *D*_*t*_ (*t* = 1, 2, 3): less than 10 min, between 10 and 20 min, and between 20 and 30 min. Then, it computes the supply-to-demand ratio *R*_*i*_ for each healthcare location *i*. The first step is also expressed as:

1 $${\text{R}}_{{\rm i}} = \frac{{{\text{C}}_{{\rm i}} }}{{\mathop \sum \nolimits_{{{{\rm k}} \in \left\{ {{\text{d}}_{{\rm ki}} \in {\text{D}}_{{\rm t}} } \right\}}} {\text{P}}_{{\rm k}} {\text{W}}_{{\rm t}} }}$$

where *C*_*i*_ represents the capacity of health care supply at location *i*, *P*_*k*_ represents the population size of any population location *k* within subzone *D*_*t*_, *d*_*ki*_ indicates the shortest travel time between *i* and population location *k*, and *W*_*t*_ represents the impedance weight for *D*_*t*_ based on the Gaussian function. In addition to the Gaussian function, the inverse power function and exponential function have been used in spatial interaction research [19]. However, the Gaussian function is preferred in gravity models (including the E2SFCA model) because it compares most favorably with realized access data [10, 14, 19, 46]. Choice of impedance functions and coefficients is discussed in the “Sensitivity analysis” section.

In the second step, the model sums the weighted supply-to-demand ratio of all the healthcare locations *i* within the 30-min travel-time zone of population location *j*. The second step is expressed as:

2 $${\text{A}}_{{\text{j}}}^{{\text{F}}} = \sum\limits_{{{\text{i}} \in \left\{ {{\text{d}}_{{{\text{ji}}}} \in {\text{D}}_{{\text{t}}} } \right\}}} {{\text{R}}_{{\text{i}}} } {\text{W}}_{{\text{t}}}$$

where $A_{j}^{F}$ is the spatial access index for any population location *j*, $R_{i}$ represents the supply-to-demand ratio for any healthcare location *i* within the 30-min travel-time zone of each population location *j*, and *ji* represents the shortest travel time between *j* and *i*.

The uncertainty produced by the different impedance coefficients in the E2SFCA method was noted and a relative spatial access measurement method [42] was developed to address this issue. In the relative spatial access method, the concept of spatial access ratio (SPAR) was introduced to describe levels of relative spatial access based on the E2SFCA method. Specifically, it first computes a spatial access index (SPAI; indicated by $A_{j}^{F}$ in Eq. 2) for each population location using the E2SFCA method, then calculates a ratio of SPAI in each population location to the average SPAI of the entire region to represent the level of relative spatial access. Sensitivity analysis results suggest that SPAR is stable and not sensitive to the choice of impedance coefficient while the SPAI varies significantly under different coefficients.

### Multi-modal relative spatial access assessment approach

Our proposed approach is implemented in the following 3 steps:

*Step 1*: Calculate the supply-to-demand ratio for each PCP location:

3 $$R_{i} = \frac{{C_{i} }}{{\mathop \sum \nolimits_{{k \in d_{{ki,M_{1} }} \le d_{{1,M_{1} }} }} P_{{k,M_{1} }} *W_{ki } + \mathop \sum \nolimits_{{k \in d_{{ki,M_{2} }} \le d_{{2,M_{2} }} }} P_{{k,M_{2} }} *W_{ki } + \cdots + \mathop \sum \nolimits_{{k \in d_{{ki,M_{n} }} \le d_{{n,M_{n} }} }} P_{{k,M_{n} }} *W_{ki } }}$$

where $\mathop \sum \nolimits_{{k \in d_{{ki,M_{n} }} \le d_{{n,M_{n} }} }} P_{{k,M_{n} }} *W_{ki } = \mathop \sum \nolimits_{{k \in \left\{ {d_{{ki,M_{n} }} \in D_{{1,M_{n} }} } \right\}}} P_{{k,M_{n} }} *W_{{Z_{1} }} + \mathop \sum \nolimits_{{k \in \left\{ {d_{{ki,M_{n} }} \in D_{{2,M_{n} }} } \right\}}} P_{{k,M_{n} }} *W_{{Z_{2} }} + \cdots + \mathop \sum \nolimits_{{k \in \left\{ {d_{{ki,M_{n} }} \in D_{{n,M_{n} }} } \right\}}} P_{{k,M_{n} }} *W_{{Z_{n} }}$ and where *R*_*i*_ is the supply-to-demand ratio for each healthcare location *i*, the denominator is the demand population, composed of populations under different transportation modes. *C*_*i*_ represents the capacity of health care supply at location *i*, $P_{k, Mn}$ represents the population size of any population location *k* within the travel mode $M_{n}$ catchment of healthcare location $i$ defined by $d_{{ki,M_{n} }} \le d_{{n,M_{n} }}$, where $d_{{ki,M_{n} }}$ is the travel time between population location $k$ and healthcare location $i$ under travel mode $M_{n}$, and $d_{{n,M_{n} }}$ is the specified threshold travel time for travel mode $M_{n}$. $W_{ki}$ represents the impedance weight (how travel cost impacts accessibility) based on the Gaussian function $W_{ki} = e^{{ - d^{2} /\beta }} , {\text{where}} \beta$ is an impedance coefficient. *Z*_*n*_ represents subzone *n*; each subzone is defined by a threshold travel time *D*_*n*_ and weights are equal within each subzone. The mean travel time in each subzone was used to calculate the respective weight.

*Step 2*: Calculate the travel mode-specific spatial access index (SPAI) and an integrated SPAI for each population location:

4 $$\begin{aligned} A_{j} & = A_{{j, M_{1} }} + A_{{j, M_{2} }} + \cdots + A_{{j, M_{n} }} \\ & = \mathop \sum \limits_{{i \in d_{{ji,M_{1} }} \le d_{{1,M_{1} }} }} R_{i} *W_{ji } + \mathop \sum \limits_{{i \in d_{{ji,M_{2} }} \le d_{{2,M_{2} }} }} R_{i} *W_{ji } + \cdots + \mathop \sum \limits_{{i \in d_{{ji,M_{n} }} \le d_{{n,M_{n} }} }} R_{i} *W_{ji } \\ A_{{j, M_{n} }} & = \mathop \sum \limits_{{i \in d_{{ji,M_{n} }} \le d_{{n,M_{n} }} }} R_{i} *W_{ji } \\ \end{aligned}$$

where

$$\mathop \sum \limits_{{i \in d_{{ji,M_{n} }} \le d_{{n,M_{n} }} }} R_{i} *W_{ji } = \mathop \sum \limits_{{k \in \left\{ {d_{{ki,M_{n} }} \in D_{{1,M_{n} }} } \right\}}} R_{i} *W_{{Z_{1} }} + \, \, \mathop \sum \limits_{{k \in \left\{ {d_{{ki,M_{n} }} \in D_{{2,M_{n} }} } \right\}}} R_{i} *W_{{Z_{2} }} + \cdots + \mathop \sum \limits_{{k \in \left\{ {d_{{ki,M_{n} }} \in D_{{n,M_{n} }} } \right\}}} R_{i} *W_{{Z_{n} }}$$

and where $A_{j}$ is the integrated spatial access index (SPAI) that includes all travel modes for any population location $j$, $R_{i}$ represents the supply-to-demand ratio calculated in step 1 for any healthcare location $i$ within the travel-time zone of each population location $j$ under travel mode $M_{n}$. $A_{{j, M_{n} }}$ is the SPAI for any population location *j* under travel mode $M_{n}$. $W_{{Z_{n} }}$ represents weight in subzone *n*. This formula was adapted from a previous study [21].

We calculated results for car drivers and bus travelers separately, but these are indeed multi-modal results because they reflect that populations using different modes have different amounts of potential access to a resource. That is, the supply of PCPs is limited, so if car drivers have greater accessibility, they are reducing the supply (and therefore the accessibility) of bus riders. Or, stated more appropriately, car users have the potential to access a greater portion of the supply than do bus riders.

In our case study, step 2 (Eq. 4) becomes the following:

5 $$A_{j} = A_{{j, M_{1} }} + A_{{j, M_{2} }} = A_{j, car} + A_{j, bus}$$

where

$$A_{j, car} = \mathop \sum \limits_{{k \in \left\{ {d_{ki,car} \in (0 - 10)} \right\}}} R_{i} *W_{{Z_{1} }} + \mathop \sum \limits_{{k \in \left\{ {d_{ki,car} \in (10 - 20)} \right\}}} R_{i} *W_{{Z_{2} }} + \mathop \sum \limits_{{k \in \left\{ {d_{ki,car} \in (20 - 30)} \right\}}} R_{i} *W_{{Z_{3} }}$$

and

$$A_{j, bus} = \mathop \sum \limits_{{k \in \left\{ {d_{ki,bus} \in (0 - 10)} \right\}}} R_{i} *W_{{Z_{1} }} + \mathop \sum \limits_{{k \in \left\{ {d_{ki,bus} \in (10 - 20)} \right\}}} R_{i} *W_{{Z_{2} }} + \mathop \sum \limits_{{k \in \left\{ {d_{ki,bus} \in (20 - 30)} \right\}}} R_{i} *W_{{Z_{3} }} + \mathop \sum \limits_{{k \in \left\{ {d_{ki,bus} \in (30 - 60)} \right\}}} R_{i} *W_{{Z_{4} }}$$

and where $A_{j, car}$ and $A_{j, bus}$ (or $A_{{j, M_{1} }}$ and $A_{{j, M_{2} }}$, respectively) are mode-specific SPAI scores for subpopulations using each mode (car, bus), calculated under multi-modal access conditions, and $A_{j}$ is the integrated access score for the whole population and both modes. The catchment size for cars, or $d_{{1,M_{1} }}$, is set at 30 min and the catchment size for bus travel, or $d_{{2,M_{2} }}$, is set at 60 min. Three subzones (0 to 10 min, 10 to 20 min, and 20 to 30 min) are defined for car travelers. Four subzones (0 to 10 min, 10 to 20 min, 20 to 30 min, and 30 to 60 min) are defined for bus riders. Weight calculations for the subzones are discussed in the “Computing access index and ratio” section.

Existing research has discussed the uncertainty brought on by the subjectivity of choosing the impedance coefficient $\beta$ [19, 42]. Wan et al. [42] developed a relative spatial assessment approach to address this limitation, which calculates a ratio between the spatial access index and the average spatial access index as the spatial access ratio (SPAR).

*Step 3*: Compute the spatial access ratio (SPAR) for each population location:

6 $$\begin{aligned} S_{j} & = A_{j} /\bar{A} \\ S_{{j, M_{n} }} & = A_{{j, M_{n} }} /\bar{A}_{{ M_{n} }} \\ \end{aligned}$$

where $S_{j}$ is the integrated SPAR, $A_{j}$ is the integrated SPAI derived from step 2, $\bar{A}$ is the average SPAI in the study area, $S_{{j, M_{n} }}$ is the SPAR for any population location j under travel mode $M_{n}$, $A_{{j, M_{n} }}$ is the SPAI for any population location under travel mode $M_{n}$, and $\bar{A}_{{ M_{n} }}$ is the average SPAI under travel mode $M_{n}$ in the study area.

SPAR has been proven to be more stable than the original SPAI when using different impedance coefficients [6, 42]. The present study expands on the concept and computes the spatial access ratio for both the combined spatial access index for all travel modes and separate spatial access index for single travel mode.

## Case study

**Fig. 1:**
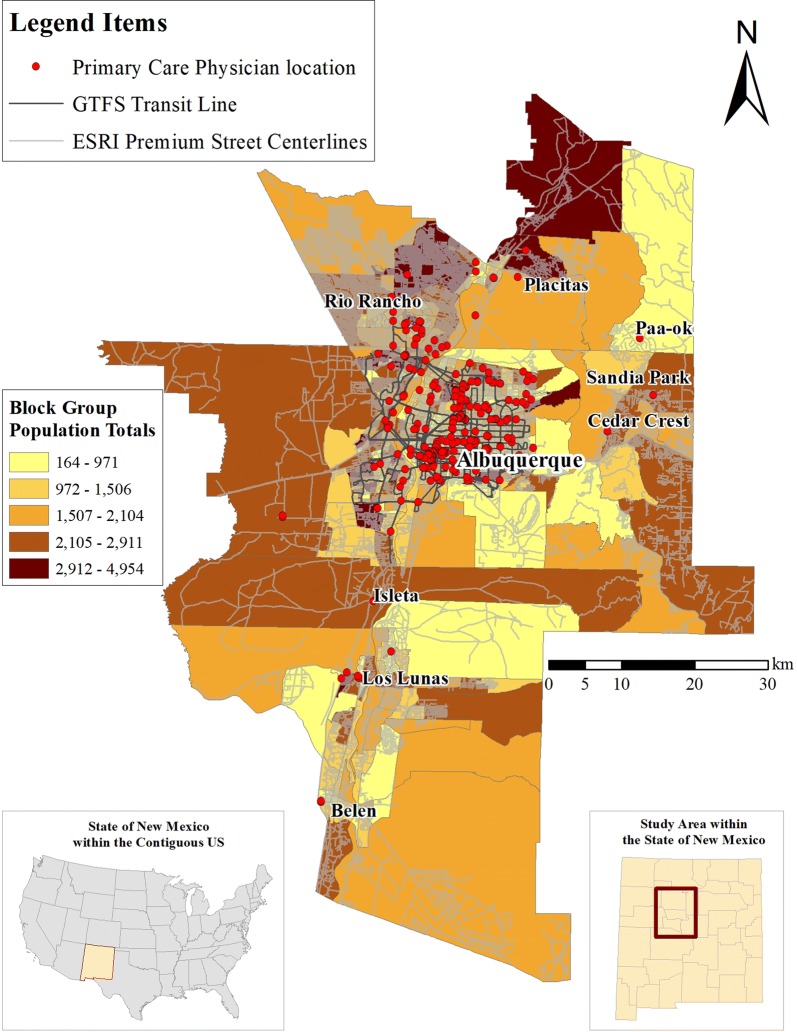
The study area of the Albuquerque Metropolitan Area in Central New Mexico

The Albuquerque metropolitan planning area was used as the study area of this study (Fig. 1), comprising Bernalillo, Sandoval, and Valencia Counties in central New Mexico, USA. Multi-modal E2SFCA results can identify areas with low spatial access to primary care, which provides information for New Mexico’s Mid-Region Council of Governments (MRCOG) for future transportation planning. The total population of this area was 868,763 as of 2015. The public transit lines (bus) are in the City of Albuquerque, near the center of the study area.

### Data

Data used in this study included: Primary care physician (PCP) location data, population counts, census block group boundaries, transportation network data, and national household travel survey data.

PCP data were obtained from the National Provider Identifier (NPI) records [29]. We selected NPI records that indicate a specialty of family practice, family medicine, general practice, general pediatrics, or general internal medicine. Because of well-known data limitations in NPI data (e.g., incorrect practicing addresses), we also collected data on family practice physicians from the Infogroup—an Esri business partner. The data was purchased by the New Mexico Department of Information Technology (DOIT) who provided it to the New Mexico Community Data Collaborative (NMCDC), managed by the New Mexico Department of Health (NMDOH) [13]. We first merged the NPI and Infogroup databases and removed duplicate records. We then conducted a data validation process to validate or correct the practicing address of PCPs through different methods, such as Google search, phone calls, and office visits. There are 1166 PCPs at 293 practicing addresses.

Population data from 2015 in 543 census block groups was obtained from the American Community Survey [40], including the total population and the percentage of people without a vehicle. The average population in each block group is approximately 1600. We then calculated the population subsets of people without a vehicle and those with at least one vehicle as a proxy of the population size for bus riders and car drivers. National household travel survey data was used to estimate the average travel time to PCPs by bus (52 min) and car (25 min) based on the average travel time for medical service related trips in New Mexico [41].

We only considered car and bus transportation modes in the present study because they are the two primary transportation modes in the study area. Theoretically, our model could include more than two transportation modes if data are available. No subway/train services are available within the metro area and walking is not practical in the study area to access PCPs. The bus mode is a combination of walking and bus riding.

We obtained ESRI premium streets network dataset to calculate travel time by car. We used General Transit Feed Specification (GTFS) data for ABQ Ride from Transitfeeds.com, which collects GTFS datasets from around the world [39] and street network data to calculate travel time by bus.

### Transportation network models for each transportation mode

#### Car

To model travel by car, we computed an origin-destination matrix from population weighted block group centroids to PCP locations using the ESRI StreetMap Premium network, which accounts for historic traffic. Population weighted block group centroids were generated based on census blocks with population attributes using the Mean Center tool in ArcMap (with block group ID as the case field (or aggregation boundary) and population as the weight field). Speed limit data and street lengths in the street dataset were used to compute the travel time of each street segment, which was used as the impedance in the street network. We modeled the connectivity of the street network, including one-way streets, turns, and overpasses/tunnels. The analysis was conducted using ArcGIS 10.4 and the Network Analyst extension.

#### Bus

**Fig. 2:**
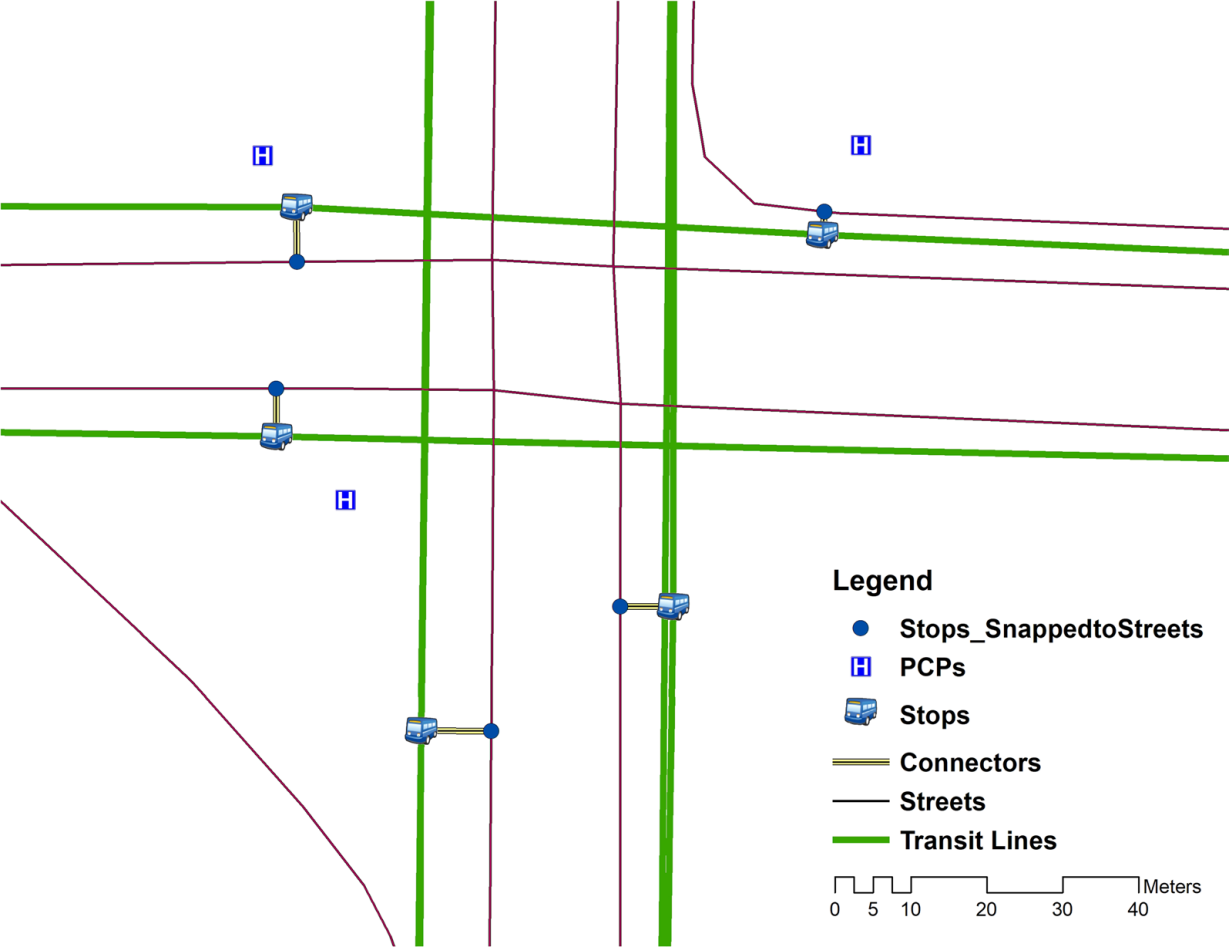
Multi-modal network

The GTFS data contains transit lines, bus stops, calendar information, as well as the arrival times at and departure times from bus stops, which are necessary to create a transit line network. ESRI streets data were also included to model pedestrians who walk between transit stops and their origins or destinations or to walk between nearby stops for transfers. We first generated feature classes for transit lines and stops and an SQL database of the schedules using the GTFS data. Then we created connector features between the transit lines and stops and streets data in the following steps: 1) A copy of transit stops was created and were snapped to the streets; 2) A line feature was created to connect the true location of each transit stop and its snapped location; and 3) Vertices were created on the street lines at the snapped location, which is necessary for establishing connectivity in the network dataset (Fig. 2).

A multi-modal network dataset was created using street lines, transit lines, transit stops, snapped locations for transit stops, and the connectors described above. A travel time cost attribute was created in the network dataset based on various sources. For example, travel time for streets was defined based on walking speed (3 miles per hour) and travel time for transit lines was based on the GTFS schedule. The analysis was conducted in ArcGIS 10.4 with the Add GTFS to a Network Dataset tool [8]. We created an origin-destination matrix using the multi-modal network dataset. Since specific dates were required to solve the network analysis, we ran the origin-destination matrix for two consecutive weeks (Monday–Friday) on an hourly basis (8 am–5 pm). The average travel time for each origin–destination pair was used in the spatial access measurement.

### Computing access index and ratio

A total of 3 groups of SPAI and SPAR were computed for each census block group using the proposed method, including spatial access for car drivers, bus riders, and integrated access. Based on the National Household Travel Survey data, we used 30 and 60 min as the thresholds (catchment sizes) for car and bus, respectively. Gaussian weights for car drivers were assigned to three subzones (0–10 min, 10–20 min, and 20–30 min). The mean travel times in each subzone (5, 15, and 25 min) were used as *d* to calculate the weight. Weights for bus riders were assigned to four subzones (0–10 min, 10–20 min, 20–30 min, and 30–60 min). The mean travel time in each subzone (5, 15, 25, and 45 min) was used as *d* to calculate the respective weight.

### Sensitivity analysis

In order to evaluate how results would be sensitive to the choice of the impedance coefficient *β*, we computed a series of SPAI and SPAR scores using different coefficients. Realized data should theoretically be used to calibrate the impedance coefficient by fitting the curve to known travel behavior, but such information is often unavailable [19, 26]. In the absence of realized data, models of potential access should not use arbitrarily assigned impedance coefficients but should assign a coefficient based on a critical weight as the function approaches 0 [19]. The critical value is usually set at or near 0.1 or 0.01 for the outermost sub-zone [19, 42, 43, 46]).

We chose 0.01 as the lowest outermost target weight; it produced a wider range of weight values across catchments at the lowest coefficient and allowed us to include weights near 0.1 in the sensitivity analysis. We identified appropriate coefficients between 140 and 320 for car drivers and coefficients between 440 and 1040 for bus riders. The minimum coefficient was based on the critical value of 0.01 and the maximum coefficient was defined as the value where the Gaussian curve started to level off. A total of 13 equal-interval coefficients each were used for car (β = 140, 155, 170, 185, 200, 215, 230, 245, 260, 275, 290, 305, 320) and bus (β = 440, 490, 540, 590, 640, 690, 740, 790, 840, 890, 940, 990, 1040). Since the denominator in $R_{i}$ in Step 1 in our method (see Eq. 3) involves weighted car travelers and bus riders, a coefficient is required for each transportation mode. We simulated 169 combinations of car and bus coefficients in the sensitivity analysis, generating 169 SPAI and SPAR scores as results. The model implementation and sensitivity analysis were conducted in ArcPy.

We used one-way ANOVA to test whether there were significant differences in the SPAI and SPAR when different impedance coefficients (β) were used. For SPAI, three null hypotheses were tested: for car, bus travel, and the integrated modes. For car, we first tested the following null hypothesis:

7 $$H_{0} {:}\,A_{SPAI, \beta = 140} = A_{SPAI, \beta = 155} = A_{SPAI, \beta = 170} = \cdots = A_{SPAI, \beta = 320} .$$

Then we tested the following null hypothesis for each coefficient (140 ≤ *β* ≤ 320) for car:

8 $$H_{0} {:}\,A_{SPAI,\beta = 440} = A_{SPAI,\beta = 490} = A_{SPAI,\beta = 540} = \cdots = A_{SPAI,\beta = 1040} .$$

For bus, we first tested the following null hypothesis:

9 $$H_{0} {:}\,A_{SPAI,\beta = 440} = A_{SPAI,\beta = 490} = A_{SPAI,\beta = 540} = \cdots = A_{SPAI,\beta = 1040} .$$

Then we tested the following null hypothesis for each coefficient (440 ≤ *β* ≤ 1040) for bus:

10 $$H_{0} {:}\,A_{SPAI, \beta = 140} = A_{SPAI, \beta = 155} = A_{SPAI, \beta = 170} = \cdots = A_{SPAI, \beta = 320} .$$

For the integrated SPAI, we first tested the following null hypothesis:

11 $$H_{0} {:}\,A_{SPAI,\beta = 140} = A_{SPAI,\beta = 155} = A_{SPAI,\beta = 170} = \cdots = A_{SPAI,\beta = 320} .$$

Then we tested the following null hypothesis for each coefficient (140 ≤ *β* ≤ 320):

12 $$H_{0} {:}\,A_{SPAI,\beta = 440} = A_{SPAI,\beta = 490} = A_{SPAI,\beta = 540} = \cdots = A_{SPAI,\beta = 1040} .$$

Similarly, we conducted three tests for SPAR. For car, we first tested the following null hypothesis:

13 $$H_{0} {:}\,A_{SPAR, \beta = 140} = A_{SPAR, \beta = 155} = A_{SPAR, \beta = 170} = \cdots = A_{SPAR, \beta = 320} .$$

Then we tested the following null hypothesis for each coefficient (140 ≤ *β* ≤ 320) for car:

14 $$H_{0} {:}\,A_{SPAR, \beta = 440} = A_{SPAR, \beta = 490} = A_{SPAR, \beta = 540} = \cdots = A_{SPAR, \beta = 1040} .$$

For bus, we first tested the following null hypothesis:

15 $$H_{0} {:}\,A_{SPAR, \beta = 440} = A_{SPAR, \beta = 490} = A_{SPAR, \beta = 540} = \cdots = A_{SPAR, \beta = 1040}$$

Then we tested the following null hypothesis for each coefficient (440 ≤ *β* ≤ 1040) for bus:

16 $$H_{0} {:}\,A_{SPAR, \beta = 140} = A_{SPAR, \beta = 155} = A_{SPAR, \beta = 170} = \cdots = A_{SPAR, \beta = 320}$$

For the integrated SPAI, we first tested the following null hypothesis:

17 $$H_{0} {:}\,A_{SPAR, \beta = 140} = A_{SPAR, \beta = 155} = A_{SPAR, \beta = 170} = \cdots = A_{SPAR, \beta = 320} .$$

Then we tested the following null hypothesis for each coefficient (140 ≤ *β* ≤ 320):

18 $$H_{0} {:}\,A_{SPAR, \beta = 440} = A_{SPAR, \beta = 490} = A_{SPAR, \beta = 540} = \cdots = A_{SPAR, \beta = 1040} .$$

## Results

Both car and bus accessibility were assessed for thirteen coefficients, but the ranges for each mode were different due to the variation in the number of subzones and the travel time of the outermost sub-zone. The coefficients, with which mode they were used, and the weights they produced in each sub-zone are detailed in Table 1. Impedance coefficients were calculated based on the target critical weight (0.01) at the mean travel time for the outermost subzone (25 min for car; 45 min for bus). The impedance coefficient was then used to calculate weights for the remaining sub-zones at their respective mean travel times.

**Table 1:** Distance impedance coefficients

Distance impedance coefficient $\varvec{\beta}$	Sub-zone 1 (0–10 min); weight at 5 min.	Sub-zone 2 (10–20 min); weight at 15 min	Sub-zone 3 (20–30 min); weight at 25 min	Sub-zone 4 (30–60 min; bus only); weight at 45 min
Car				
140	0.836	0.200	0.012	
155	0.851	0.234	0.018	
170	0.863	0.266	0.025	
185	0.874	0.296	0.034	
200	0.882	0.325	0.044	
215	0.890	0.351	0.055	
230	0.897	0.376	0.066	
245	0.903	0.399	0.078	
260	0.908	0.421	0.090	
275	0.913	0.441	0.103	
290	0.917	0.460	0.116	
305	0.921	0.478	0.129	
320	0.925	0.495	0.142	
Bus				
440	0.945	0.600	0.242	0.010
490	0.950	0.632	0.279	0.016
540	0.955	0.659	0.314	0.024
590	0.959	0.683	0.347	0.032
640	0.962	0.704	0.377	0.042
690	0.964	0.722	0.404	0.053
740	0.967	0.738	0.430	0.065
790	0.969	0.752	0.453	0.077
840	0.971	0.765	0.475	0.090
890	0.972	0.777	0.495	0.103
940	0.974	0.787	0.514	0.116
990	0.975	0.797	0.532	0.129
1040	0.976	0.805	0.548	0.143

Lower coefficients generally produced greater ranges in weights (0.935 between sub-zones 1 and 4 at β = 440), and the range decreased as the coefficient value increased (0.833 between sub-zone 1 and sub-zone 4 for bus at β = 1040). The same pattern is seen in the weights for car travel, although the change is much less pronounced (0.825 between sub-zone 1 and sub-zone 3 at β = 140; 0.783 at β = 320). In fact, the range in weights for car travel is greatest at β = 185 (0.840 between sub-zones 1 and 3). Based on these weights, we should expect two things: 1) car accessibility scores to change less than bus scores as coefficients increase; and 2) both travel modes should produce less variability in scores as coefficients increase.

### Car SPAI and SPAR

SPAI and SPAR scores were calculated for car-based travel. Table 2 lists descriptive statistics for the results, including the minimum, maximum, mean, standard deviation, and coefficient of variation (CV; standard deviation divided by the mean). The lowest impedance coefficient (β = 140) produced the highest maximum, mean, and coefficient of variation, reflecting that, as expected, the increasing coefficients created a levelling effect, reducing variation in accessibility scores and increasing accessibility across the study area. This pattern is also seen in the SPAR scores, but as is expected with SPAR, the variation is much less pronounced. Since SPAR is a ratio of a given place’s SPAI score to the average of all SPAI scores in the region, when all SPAR scores are averaged, the mean of all SPARs is always 1.0 and the variation of SPAR is small. More details about SPAR can be found elsewhere [42]. The results of the sensitivity assessment are discussed later in this section.

**Table 2:** Descriptive statistics of Spatial Access Index and Spatial Access Ratio for car travel

$\varvec{\beta}$	Car SPAI (10^−3^)					Car SPAR				
	Min	Max	Mean	SD	CV^a^	Min	Max	Mean	SD	CV^a^
140	0.000	4.570	1.667	1.155	0.693	0.000	2.731	1.000	0.693	0.693
155	0.001	4.270	1.652	1.085	0.657	0.000	2.576	1.000	0.657	0.657
170	0.001	4.025	1.639	1.029	0.628	0.000	2.448	1.000	0.628	0.628
185	0.001	3.822	1.628	0.982	0.603	0.001	2.341	1.000	0.603	0.603
200	0.001	3.652	1.619	0.943	0.583	0.001	2.250	1.000	0.583	0.583
215	0.001	3.507	1.610	0.910	0.565	0.001	2.173	1.000	0.565	0.565
230	0.001	3.383	1.602	0.880	0.549	0.001	2.106	1.000	0.549	0.549
245	0.002	3.275	1.595	0.855	0.536	0.001	2.048	1.000	0.536	0.536
260	0.002	3.180	1.589	0.832	0.524	0.001	2.047	1.000	0.524	0.524
275	0.002	3.097	1.583	0.812	0.513	0.001	1.952	1.000	0.513	0.513
290	0.002	3.022	1.578	0.793	0.503	0.001	1.912	1.000	0.503	0.503
305	0.002	2.956	1.573	0.777	0.494	0.001	1.875	1.000	0.494	0.494
320	0.002	2.896	1.569	0.761	0.485	0.001	1.843	1.000	0.485	0.485

^a^Coefficient of variation

**Fig. 3:**
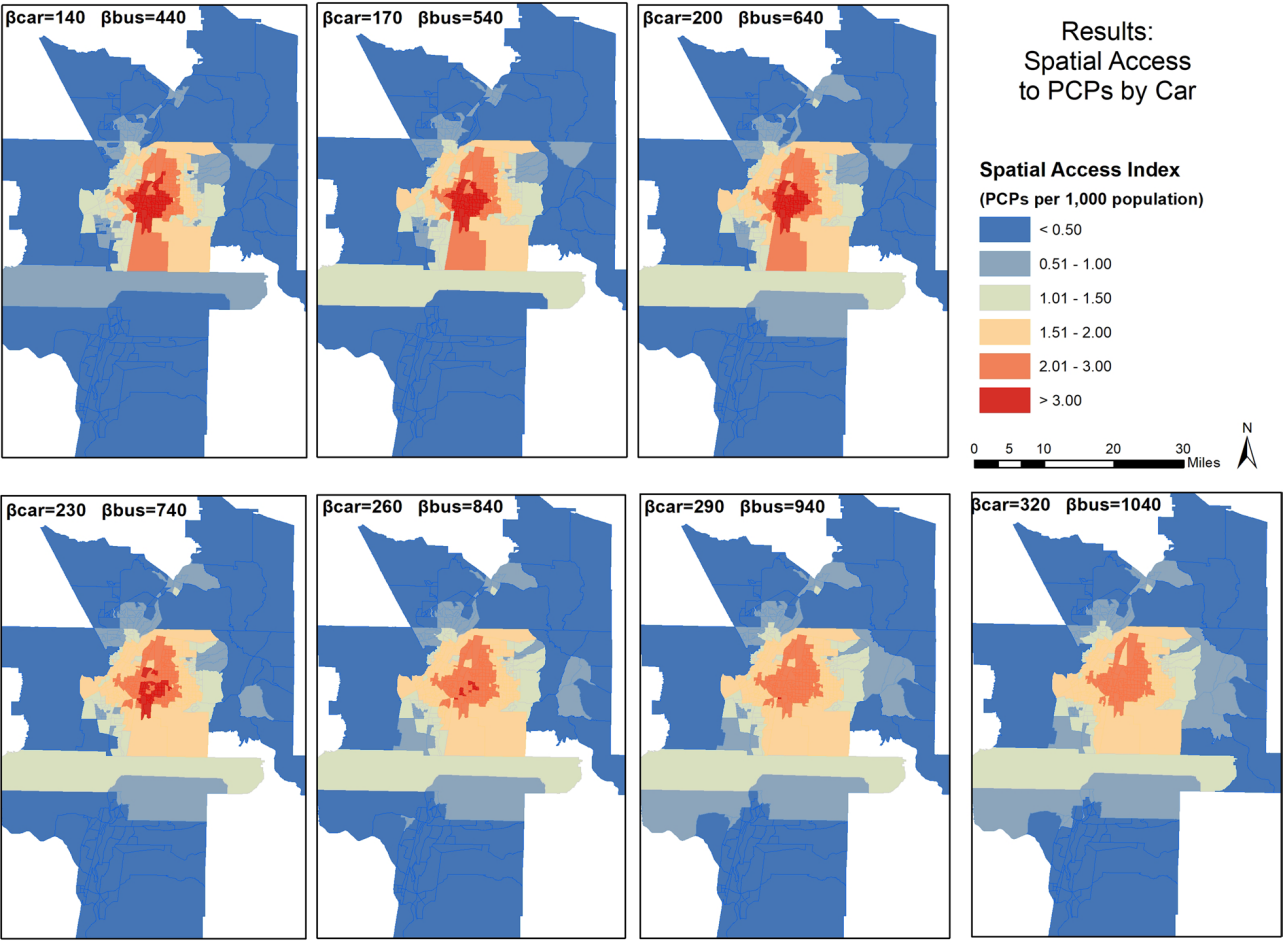
Spatial distribution of Spatial Access Index Scores for car travel in the study area

**Fig. 4:**
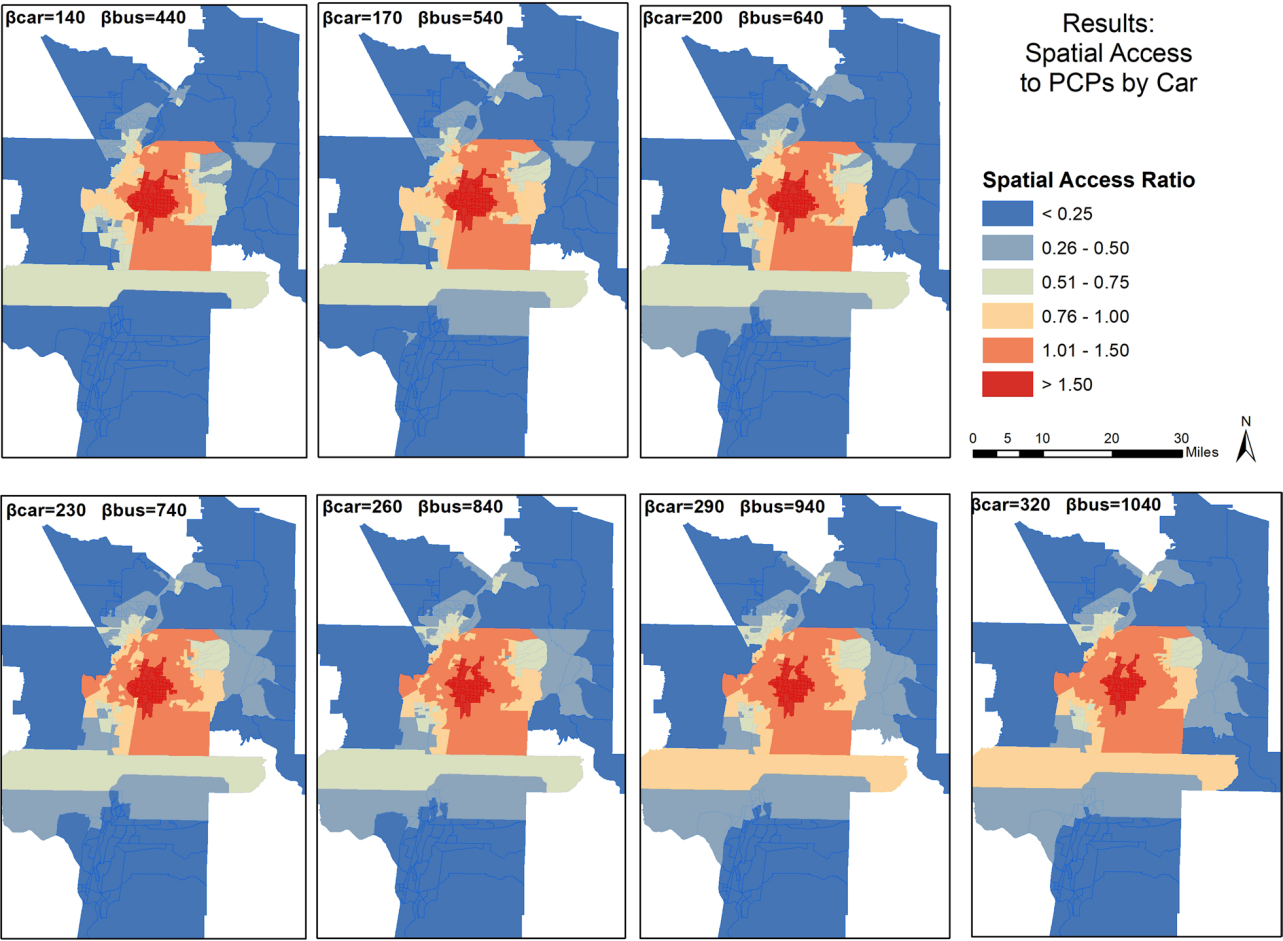
Spatial distributions of Spatial Access Ratio Scores for car travel in the study area

Figures 3 and 4 show the mapped results and geographic distribution of SPAI and SPAR scores respectively based on selected coefficients. On both sets of maps, scores are binned into six classes. For SPAI (Fig. 3), the lowest class includes all scores less than 0.50, and each subsequent class graduates by an equal interval of 0.50 until the fifth class, which ranges from 2.01 to 3.00, and then the sixth class includes all scores above 3.00. For SPAR (Fig. 4), the six classes are distributed similarly, but graduate by values of 0.25 until the fifth class, ranging from 1.01 to 1.50, and then the sixth class, for all scores above 1.50.

Across all maps in Fig. 3, higher scores are concentrated in the center of the study area, where major interstates intersect and more facilities are located. Increasing the coefficient increases the scores in outer parts of the study area and lowers the scores in the center. This is due to the populations in outer areas being modeled as exerting greater demand (via greater access) on the supply of primary care physicians, reducing the supply that is exclusive to the populations in the center of the study area. Similar patterns are evident in Fig. 4, but the changes are less pronounced, and the stabilizing effect of SPAR across coefficients is noticeable.

### Bus SPAI and SPAR

SPAI and SPAR were calculated for bus-based travel to primary care physicians in the study area. Table 3 includes descriptive statistics for the bus SPAI and SPAR scores. In contrast to car scores, the lowest coefficient for bus produced the lowest scores which increased as the coefficient increased. Similar to car scores, the coefficient of variation decreased as the coefficient increased, reflecting the steeper fall of the curve as travel time increases and the larger difference in weights across sub-zones. The SPAI scores for bus are considerably lower than the car scores. Although the bus SPAR scores are more consistent across different coefficients, they show the dramatic extent of the heterogeneity of the scores within a single coefficient: the maximum scores show that some areas have over sixteen times the average access. This is likely due to outer regions of the study area having very limited or no accessibility by bus within the threshold travel time.

**Table 3:** Descriptive Statistics of Spatial Access Index and Spatial Access Ratio for Bus Travel

$\varvec{\beta}$	Bus SPAI (10^−3^)					Bus SPAR				
	Min	Max	Mean	SD	CV^a^	Min	Max	Mean	SD	CV^a^
440	0.000	0.472	0.019	0.041	2.138	0.000	16.782	1.000	2.138	2.138
490	0.000	0.512	0.024	0.047	1.954	0.000	14.494	1.000	1.954	1.954
540	0.000	0.552	0.030	0.054	1.804	0.000	12.681	1.000	1.804	1.804
590	0.000	0.591	0.036	0.061	1.684	0.000	11.250	1.000	1.684	1.684
640	0.000	0.630	0.043	0.068	1.591	0.000	10.119	1.000	1.591	1.591
690	0.000	0.669	0.050	0.076	1.517	0.000	9.218	1.000	1.517	1.517
740	0.000	0.707	0.057	0.084	1.460	0.000	8.493	1.000	1.460	1.460
790	0.000	0.746	0.065	0.092	1.414	0.000	7.904	1.000	1.414	1.414
840	0.000	0.784	0.072	0.100	1.383	0.000	7.436	1.000	1.383	1.383
890	0.000	0.821	0.081	0.109	1.348	0.000	7.018	1.000	1.348	1.348
940	0.000	0.858	0.089	0.117	1.324	0.000	6.680	1.000	1.324	1.324
990	0.000	0.894	0.097	0.126	1.304	0.000	6.394	1.000	1.304	1.304
1040	0.000	0.930	0.104	0.135	1.288	0.000	6.150	1.000	1.288	1.288

^a^Coefficient of variation

**Fig. 5:**
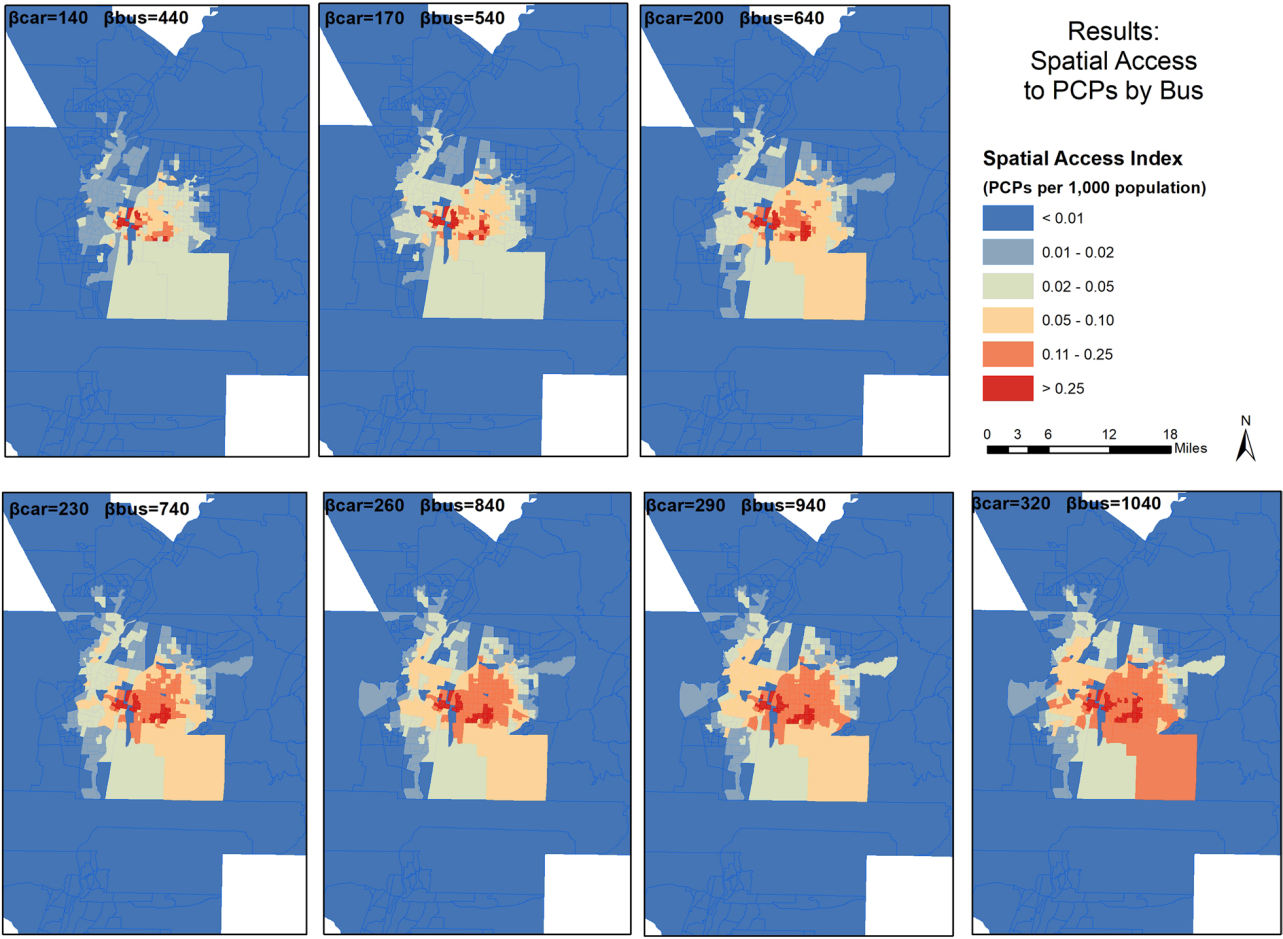
Spatial distribution of Spatial Access Index Scores for bus travel in the study area

**Fig. 6:**
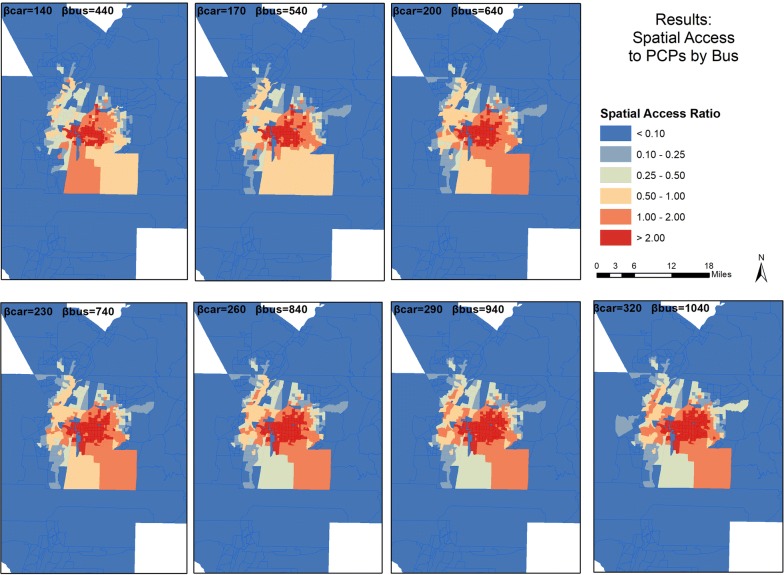
Spatial distribution of Spatial Access Ratio Scores for bus travel in the study area

These observations are reflected in the geographic distribution in the mapped results (Figs. 5, 6). The bus SPAI scores (Fig. 5) were binned into six classes: less than 0.01, 0.01–0.02, 0.02–0.05, 0.05–0.10, 0.11–0.25, and greater than 0.25. The highest scores are in the center of the study area, where bus travel options are the greatest. However, the high score areas include noticeably fewer block groups than in the car analysis. The variation as the coefficients increase is apparent, with the east and southeast regions of the study area gaining the most accessibility.

Figure 6 presents the spatial distributions of SPAR scores, also binned into six classes: less than 0.10, 0.10–0.25, 0.25–0.50, 0.50–1.00, 1.00–2.00, and greater than 2.00. While the south-center of the study area loses some accessibility as the coefficients increase, the SPAR scores overall show more stable results. However, it is clear that bus travel greatly limits accessibility outside the center of the study area and limits the populations’ abilities to exert demand on the supply of physicians.

### Integrated SPAI and SPAR

Finally, integrated SPAI and SPAR scores were calculated, representing combined access scores for all populations within a census block. Table 4 lists descriptive statistics for the results, which are itemized by the car and bus coefficients. For example, for the row of $\beta$ car = 140, the statistics describe all combinations of bus coefficients (from $\beta$ car = 140, $\beta$ bus = 440 to $\beta$ car = 140, $\beta$ bus = 1040). In these integrated scenarios, increasing the car-mode impedance coefficient ($\beta$) increases the minimum scores, but decreases the maximum, mean, and standard deviation, reflecting the homogenizing effect seen in the car-mode SPAI results. Bus-mode integrated SPAI, on the other hand, maintains the opposite pattern, as seen in the previous bus-mode results. The maximum, mean, standard deviation, and coefficient of variation all increase as bus impedance coefficients increase. Car- and bus-mode SPAR results show lower maximum scores and standard deviations that are equal to the coefficient of variation.

**Table 4:** Descriptive Statistics of Integrated Spatial Access Index and Spatial Access Ratio scores

$\varvec{\beta}$	Integrated SPAI (10^−3^)					Integrated SPAR				
	Min	Max	Mean	SD	CV^a^	Min	Max	Mean	SD	CV^a^
Car										
140	0.000	5.009	1.753	1.243	0.709	0.000	2.773	1.000	0.709	0.709
155	0.001	4.680	1.730	1.165	0.673	0.000	2.627	1.000	0.673	0.673
170	0.001	4.411	1.711	1.102	0.644	0.000	2.507	1.000	0.643	0.643
185	0.001	4.188	1.695	1.049	0.619	0.001	2.406	1.000	0.619	0.619
200	0.001	4.000	1.681	1.005	0.598	0.001	2.321	1.000	0.598	0.598
215	0.001	3.841	1.669	0.968	0.580	0.001	2.247	1.000	0.579	0.579
230	0.001	3.703	1.658	0.935	0.564	0.001	2.183	1.000	0.563	0.563
245	0.002	3.583	1.648	0.906	0.550	0.001	2.127	1.000	0.549	0.549
260	0.002	3.478	1.640	0.881	0.537	0.001	2.077	1.000	0.537	0.537
275	0.002	3.385	1.632	0.858	0.526	0.001	2.032	1.000	0.525	0.525
290	0.002	3.302	1.625	0.837	0.515	0.001	1.992	1.000	0.515	0.515
305	0.002	3.228	1.618	0.819	0.506	0.001	1.956	1.000	0.506	0.506
320	0.002	3.161	1.612	0.802	0.497	0.001	1.924	1.000	0.497	0.497
Bus										
440	0.000	4.670	1.631	0.942	0.578	0.000	2.745	1.000	0.574	0.574
490	0.000	4.691	1.636	0.947	0.579	0.000	2.747	1.000	0.575	0.575
540	0.000	4.724	1.641	0.952	0.580	0.000	2.755	1.000	0.576	0.576
590	0.000	4.758	1.646	0.957	0.581	0.000	2.762	1.000	0.578	0.578
640	0.000	4.790	1.652	0.963	0.583	0.000	2.766	1.000	0.579	0.579
690	0.000	4.822	1.659	0.969	0.584	0.000	2.770	1.000	0.580	0.580
740	0.000	4.852	1.665	0.975	0.586	0.000	2.772	1.000	0.581	0.581
790	0.000	4.881	1.672	0.982	0.587	0.000	2.773	1.000	0.583	0.583
840	0.000	4.908	1.679	0.988	0.588	0.000	2.772	1.000	0.584	0.584
890	0.000	4.935	1.686	0.995	0.590	0.000	2.772	1.000	0.585	0.585
940	0.000	4.961	1.694	1.001	0.591	0.000	2.770	1.000	0.587	0.587
990	0.000	4.986	1.701	1.008	0.593	0.000	2.768	1.000	0.588	0.588
1040	0.000	5.009	1.708	1.015	0.594	0.000	2.766	1.000	0.589	0.589

^a^Coefficient of variation

**Fig. 7:**
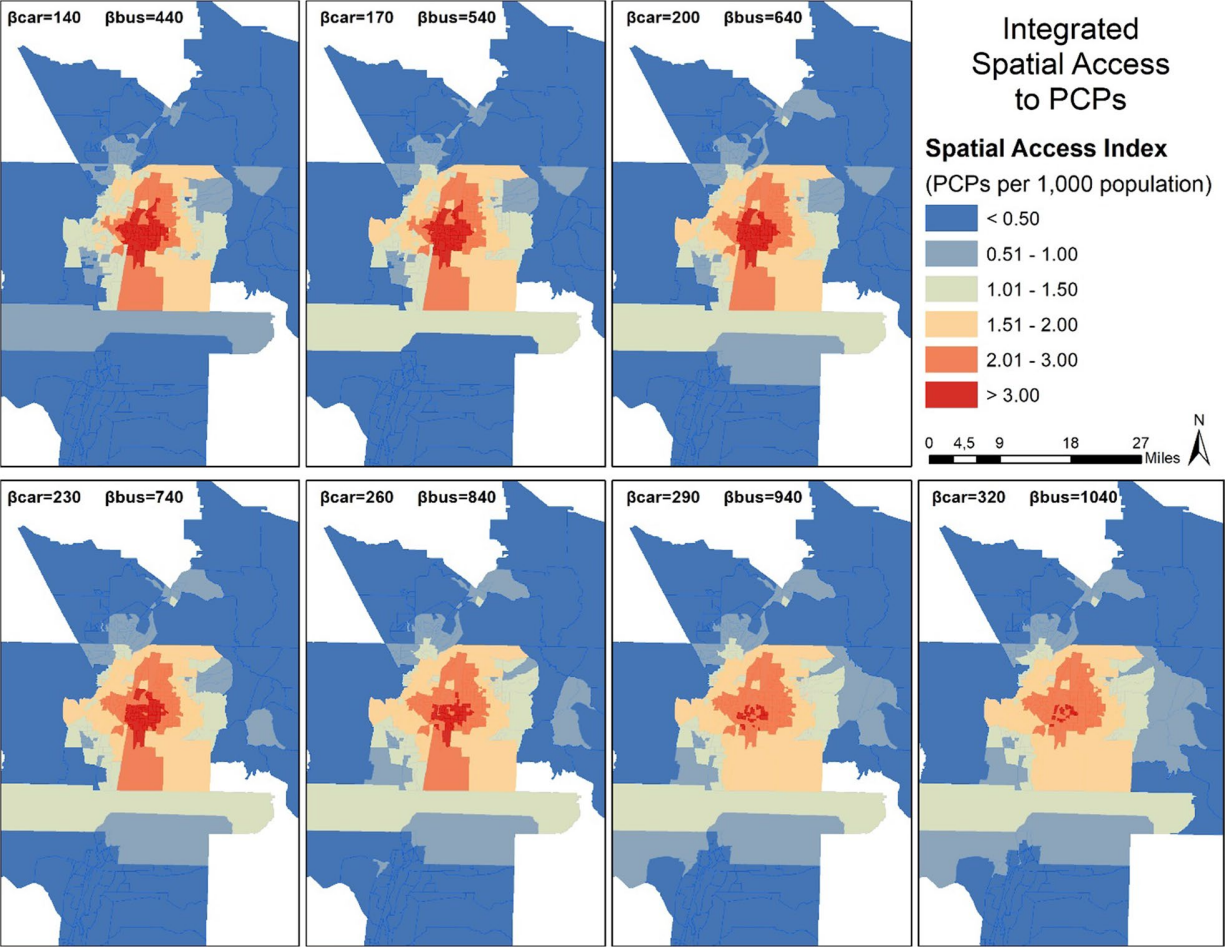
Spatial distribution of Integrated Spatial Access Index (SPAI) scores for both car and bus travel in the study area

**Fig. 8:**
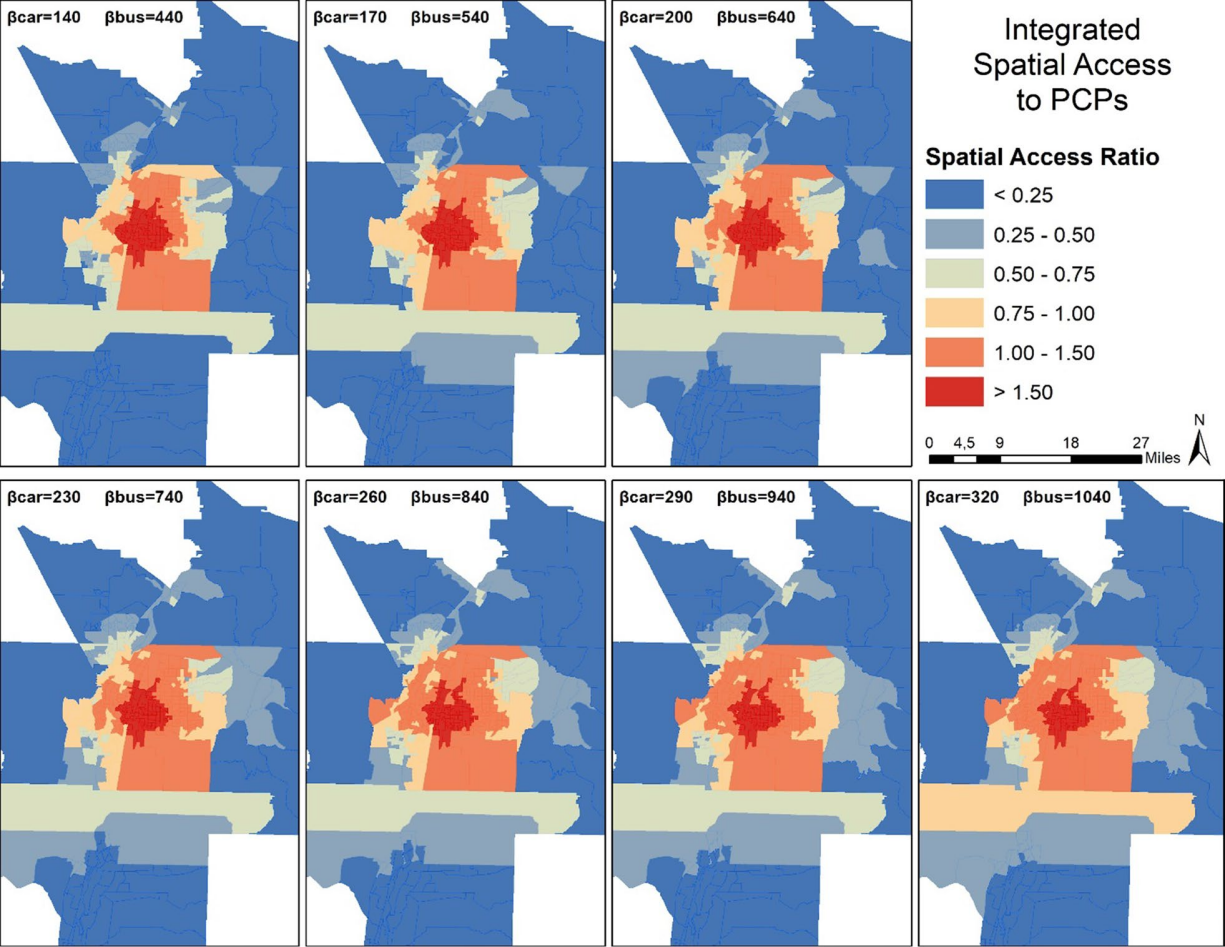
Spatial distribution of Integrated Spatial Access Ratio (SPAR) scores for both car and bus travel in the study area

Figures 7 and 8 present the mapped scores for integrated SPAI and integrated SPAR, respectively. Figure 7 shows that the integrated method does not change the general pattern of spatial distribution, with high scores in the center of the study area and low scores outside the most urban areas. In both figures, both car and bus impedance coefficients increase incrementally from top left to bottom right. The top left map has the lowest coefficient for each mode, while the bottom right features the highest coefficient for each mode. As coefficients increase for SPAI, the center areas lose access while the periphery gains, leading to a homogenizing effect within the most urban areas. However, the SPAR scores again remain more stable, with the center of the urban area featuring a large patch of high scores regardless of coefficient.

### Sensitivity analysis

**Fig. 9:**
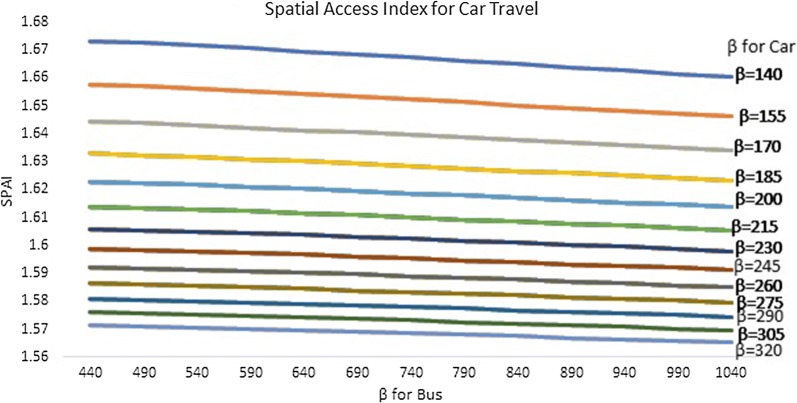
Sensitivity analysis results of Spatial Access Index for car travel

Figure 9 presents the sensitivity analysis results for spatial access index for car travel. Since SPAI was calculated using a multi-modal method with different combinations of coefficients for car and bus travel, each line represents the mean SPAI using a specific coefficient (*β*) for car travel. Different SPAI values within each line represent the mean value using different coefficients for bus travel. For example, the line of β = 140 (the top blue line) represents all of the mean SPAI scores based on the following combination of coefficients from left to right: β_car_ = 140, β_bus_ = 440; β_car_ = 140, β_bus_ = 490; β_car_ = 140, β_bus_ = 540; β_car_ = 140, β_bus_ = 590; β_car_ = 140, β_bus_ = 640; β_car_ = 140, β_bus_ = 690; β_car_ = 140, β_bus_ = 740; β_car_ = 140, β_bus_ = 790; β_car_ = 140, β_bus_ = 840; β_car_ = 140, β_bus_ = 890; β_car_ = 140, β_bus_ = 940; β_car_ = 140, β_bus_ = 990; and β_car_ = 140, β_bus_ = 1040.

We noticed that SPAI for car travel decreased as the coefficient for car travel increased from 140 to 320. SPAI also decreased as the coefficient for bus travel (x-axis) increased from 440 to 1040, although it is less pronounced.

**Fig. 10:**
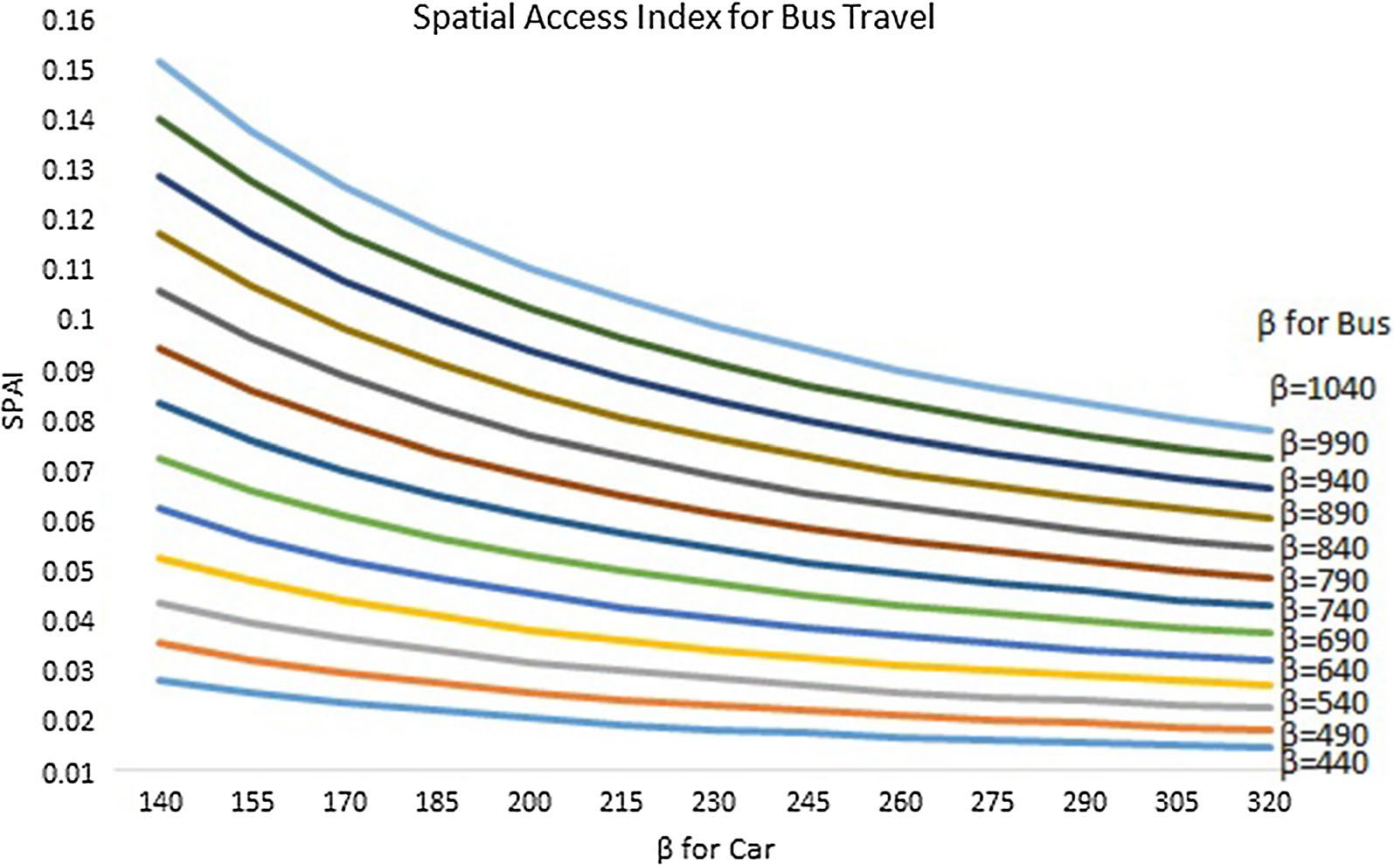
Sensitivity analysis Results of Spatial Access Index for bus travel

Figure 10 presents the sensitivity analysis results for spatial access index scores for bus travel. Each line represents the mean SPAI using a specific coefficient for bus travel. Different SPAI values within each line represent the mean value using different coefficient for car mode. For example, the line of β = 1040 (the top blue line) represents all of the mean SPAI based on the following combination of coefficients from left to right: β_bus_ = 1040, β_car_ = 140; β_bus_ = 1040, β_car_ = 155; β_bus_ = 1040, β_car_ = 170; β_bus_ = 1040, β_car_ = 185; β_bus_ = 1040, β_car_ = 200; β_bus_ = 1040, β_car_ = 215; β_bus_ = 1040, β_car_ = 230; β_bus_ = 1040, β_car_ = 245; β_bus_ = 1040, β_car_ = 260; β_bus_ = 1040, β_car_ = 275; β_bus_ = 1040, β_car_ = 290; β_bus_ = 1040, β_car_ = 305; and β_bus_ = 1040, β_car_ = 320.

We noticed that SPAI for bus travel increased as the coefficient for bus travel increased from 440 to 1040. However, SPAI decreased significantly as the coefficient for car travel (x-axis) increased from 140 to 320.

**Fig. 11:**
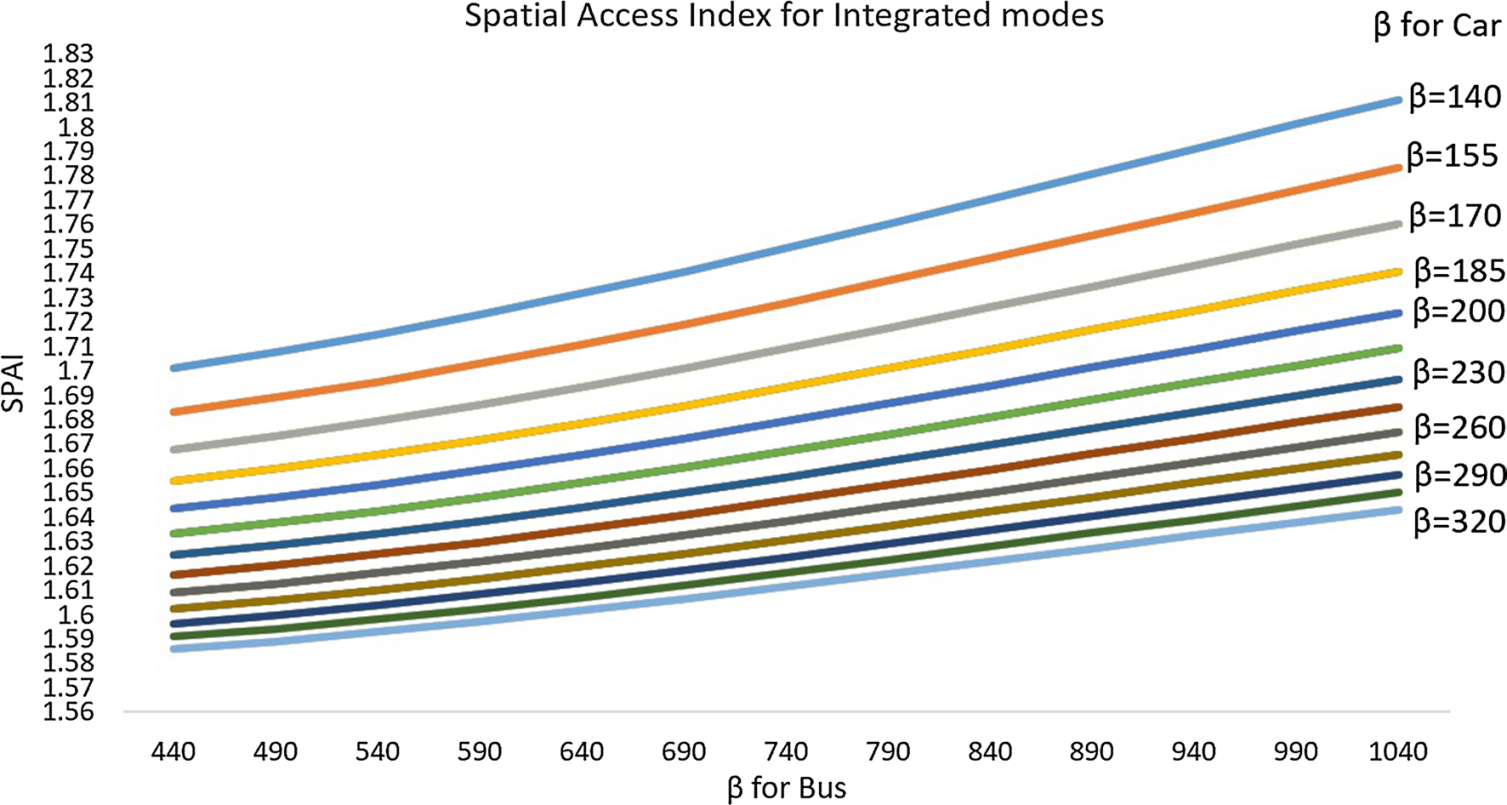
Sensitivity analysis results of integrated (bus and car) Spatial Access Index

Finally, Fig. 11 presents the sensitivity analysis results for the integrated spatial access index scores. Each line represents the mean SPAI using a specific coefficient for car travel. Different SPAI values within each line represent the mean value using different coefficients for bus travel. For example, the line of β = 320 (the bottommost, light blue line) represents all of the mean integrated SPAI scores based on the following combination of coefficients, from left to right: β_car_ = 320, β_bus_ = 440; β_car_ = 320, β_bus_ = 490; β_car_ = 320, β_bus_ = 540; β_car_ = 320, β_bus_ = 590; β_car_ = 320, β_bus_ = 640; β_car_ = 320, β_bus_ = 690; β_car_ = 320, β_bus_ = 740; β_car_ = 320, β_bus_ = 790; β_car_ = 320, β_bus_ = 840; β_car_ = 320, β_bus_ = 890; β_car_ = 320, β_bus_ = 940; β_car_ = 320, β_bus_ = 990; and β_car_ = 320, β_bus_ = 1040.

We noticed that the integrated SPAI decreased as the coefficient for car travel increased from 140 to 320, which is similar to the pattern revealed in the car-only mode (Fig. 9). However, the integrated SPAI increased as the coefficient for bus travel (x-axis) increased from 440 to 1040, due to the addition of car-only and bus-only SPAI.

A one-way ANOVA test revealed significant variations in SPAI across different coefficients (440 ≤ *β* ≤ 1040) ($p < 0.0001$) for bus-only travel (Hypothesis 9). Also, within the same group of coefficients for bus travel (440 ≤ *β* ≤ 1040), significant variations exist in SPAI when different coefficients for car were used ($p < 0.0001$) (Hypothesis 10). For car travel, although we found significant variations in SPAI across different coefficients (140 ≤ *β* ≤ 320) ($p$ < 0.05) (Hypothesis 7), SPAI did not vary significantly within the same group of car coefficients (p = 0.1) (Hypothesis 8). For integrated results, a one-way ANOVA test revealed significant variations in SPAI across different coefficients (140 ≤ *β* ≤ 320) (p < 0.0001) (Hypothesis 11). Within the same group of coefficients for car travel, significant variations also exist in SPAI when different coefficients for bus were used (p < 0.0001) (Hypothesis 12).

For SPAR, the one-way ANOVA test found no significant variations across different coefficients for hypothesis 13 through 18 ($p$ = 1). To summarize, the sensitivity analysis indicated that SPAI varies significantly while the SPAR remains stable for car travelers, bus riders, and integrated score when different coefficients were used.

## Discussions and conclusions

The E2SFCA method has been widely implemented for measuring spatial accessibility to health resources. However, the use of the impedance coefficient in an impedance function (e.g., the Gaussian function) introduces uncertainty to E2SFCA as access scores may change significantly as impedance coefficients change. Under the multi-modal framework, this paper proposed an enhancement to the E2SFCA methods by incorporating a spatial access ratio for spatial access measurement. To provide a baseline for comparison, we also evaluated spatial access using the single-modal E2SFCA (car only) based on impedance coefficients of 140–320 for car travelers. Consistent with previous findings [21], we also found that spatial access indices with car-only travel (mean 1.456–1.542) are lower (p < 0.05) than the spatial access index for car travelers using the multi-modal method (mean 1.569–1.667). Since the single-modal E2SFCA was not the focus of the present study and previous studies have discussed the differences between the single-modal and multi-modal methods, the full results of the single-modal method were not presented here. Consistent with previous research, SPAI for bus riders was significantly lower compared with car drivers. This was due to less population competing with car travelers within each catchment when the entire demanding population was subdivided into car drivers and bus riders using the multi-modal approach. Put another way, bus riders have less opportunity to exert demand on a supply or resource. Therefore, single-modal methods tend to overestimate the overall spatial accessibility for population who rely on other transportation modes that are slower than car (e.g., bus).

Our previous study has demonstrated the variation in SPAI using the single-modal (car only) E2SFCA method [42]. Our sensitivity analysis results in the present study suggest that the SPAI also varied significantly when different Gaussian impedance coefficients were used. This finding has important methodological implications, since previous studies suggest the Gaussian curve is the preferred travel impedance function in the gravity model [19]. When the Gaussian function is used, variability due to the choice of coefficient (β) is even more crucial for multi-modal E2SFCA because of the inclusion of multiple transportation modes which might be associated with different patterns of travel impedance. That is, different travel modes may see their access, or ability to exert demand, decrease with distance at a different rate. We found that SPAI for car travel is not sensitive to the coefficient for bus (average change in SPAI as coefficient for bus increases: − 0.53% based on results presented in Fig. 9). However, SPAI for bus is greatly impacted by coefficient for car with an average change in SPAI of − 48.65% as the coefficient for car increases based on results presented in Fig. 10. This observation could be explained by the disproportionately higher percentage of car travelers compared with bus riders in the study area.

We found that SPAI decreases as the impedance coefficient (*β*) increases for car mode, while SPAI increases as *β* increases for bus mode. Overall, the *R* value (supply-to-demand ratio) calculated in Eq. 3 decreases as *β* increases since the weight increases and thus the denominator increases. However, different patterns were observed in SPAI for cars and buses due to the different extents to which the denominator in Eq. 3 increases, which is primarily attributed to unbalanced distribution of population under these two transportation modes (with a higher percentage of car drivers and low percentage of bus riders). For car-specific SPAI, the increase in the denominator and thus the decrease in *R* value in Eq. 3 is stronger than the increase in the weight when calculating the SPAI in Eq. 4, which makes the SPAI smaller as *β* increases. However, for bus-specific SPAI, the increase in the denominator and thus the decrease in *R* value in Eq. 3 is weaker than the increase in the weight when calculating the SPAI in Eq. 4, which makes the SPAI larger as *β* increases. The integrated SPAI has a different pattern, in which scores decreased as the coefficient for car travel increased and scores increased as the coefficient for bus travel increased. This is primarily due to the fact that the integrated SPAI is the sum of car-specific SPAI and bus-specific SPAI.

Although SPAI, which represents the ratio of the number of PCPs to the demanding population, can be used to monitor compliance with national guidelines on social equity (e.g., 0.79 PCPs per 1000 population as the national benchmark) and identify healthcare shortage areas, the extent to which it is subject to the impedance coefficient cannot be ignored, especially when the multi-modal E2SFCA is used. SPAR, on the other hand, demonstrated significant stability with the different impedance coefficients used in the model despite its mathematical simplicity. It is a suitable alternative for SPAI for mapping purposes and analyses that do not require an absolute measure of supply to demand ratio [42].

The multi-modal FCA approach is generally flexible in defining different travel-time thresholds for different transportation modes [21]. In this study, we used different travel time tolerances for car (30 min) and bus (60 min), assuming that bus riders are willing to travel longer distances to seek healthcare and considering that the average travel time to medical services is 52 min by public transportation in New Mexico. Future studies could extend the time threshold for car travel to 60 min and examine the sensitivity of SPAI. The same set of coefficients for bus could therefore be applied to car travel: 440, 490, 540, 590, 640, 690, 740, 790, 840, 890, 940, 990, 1040. We expect to see a greater sensitivity in SPAI for both bus and car modes due to a higher variability in the Gaussian weight associated with the new coefficients.

We observed that the SPAI scores for bus riders were very low compared with the car SPAI, which was primarily due to the inconvenient public transit service that was limited to the core area of the Albuquerque metropolitan area. The SPAI for bus could not match that for car even if the travel time threshold was set to 90 min for bus. It might be worthwhile to apply our method in an area with more convenient public transportation. We examined the data in the present study and found 55% of the census block groups are beyond 30 min travel by bus to the closest primary care physician. For the remaining 45% of census block groups within 30 min bus travel to the closest PCP, each block group could access an average of only 6.5 PCP locations (out of 293 in the study area) within the 30-min threshold. Another possible explanation is that we used population-weighed census block group centers as the origin, which might lead to a longer travel time by bus than an actual residence location. Future studies might consider using a fine-scale grid network if population data are available.

Compared with other multi-modal methods based on the FCA framework, the proposed method has the following strengths. First, we applied a Gaussian impedance function in the multi-modal E2SFCA method. Previous multi-modal E2SFCA methods only used linear distance decay. However, the Gaussian function is preferred in gravity models because it compares most favorably with realized access data [10, 14, 19, 46]. Second, we evaluated the impact of different Gaussian coefficients on SPAI. An exhaustive simulation was conducted based on different combinations of coefficients. This is the first study to evaluate the sensitivity of Gaussian impedance coefficients in a multi-modal framework. Third, we introduced SPAR as an alternative measurement for multi-modal spatial accessibility when the Gaussian function is used. SPAR has been proven more reliable in single-modal E2SFCA methods, and this is the first study to prove that SPAR is also effective in a multi-modal framework.

This study is subject to several limitations. The first limitation is related to data accuracy issues in the PCP data. Our PCP data were primarily based on the NPI licensure data, which would result in incorrect practicing addresses when professionals used a residential address to obtain licensure rather than a practice address. Although we used Infogroup data and validated and updated PCPs practicing addresses in the current study, future studies should combine different sources of PCP data (e.g., license renewal survey data) for more accurate PCP data. Second, we used administrative units and created choropleth maps rather than continuous surfaces for the SPAI and SPAR. These units are indeed prone to the modifiable areal unit problem (MAUP), but they also offer opportunities for using census data (e.g., population with/without vehicle) which is necessary for the present study. Since this manuscript focuses on the methodology of comparing SPAR against SPAI for multi-model E2SFCA, we feel that using administrative boundaries for visualization is more straightforward for readers to understand the methodology. However, producing continuous accessibility surface might be ideal for cartographic representation. We suggest that future studies use continuous surfaces when adopting the present method. Third, we used GTFS data to generate travel time by bus, which is subject to the date/time schedule and traffic. We averaged a 2-week travel time data in the present study, so temporal change was not considered. Future studies could evaluate the temporal pattern of SPAI and SPAR (e.g., the effect of rush hour) based on GTFS data. Fourth, while the multi-modal FCA approach is generally flexible to define different travel-time thresholds for different transportation modes, we did not examine the impact of using other travel-time thresholds (e.g., 60 min for car) or using different subzones. For example, instead of using 0–10, 10–20, 20–30, and 30–60 min sub-zones, future studies could examine 0–15, 15–30, 30–45, and 45–60 min sub-zones for bus. Lastly, we only used the distance/travel time impedance for the impedance function in the model. However, in reality, patients’ willingness to access PCPs is impacted by many factors, such as the perceived quality of PCPs, insurance restrictions, languages, waiting time, and others. Future studies should model the impedance function based on more realistic conditions and realized data, where it is available.
